# Understanding of multi-level resistive switching mechanism in GeO_x_ through redox reaction in H_2_O_2_/sarcosine prostate cancer biomarker detection

**DOI:** 10.1038/s41598-017-11657-4

**Published:** 2017-09-11

**Authors:** Subhranu Samanta, Sheikh Ziaur Rahaman, Anisha Roy, Surajit Jana, Somsubhra Chakrabarti, Rajeswar Panja, Sourav Roy, Mrinmoy Dutta, Sreekanth Ginnaram, Amit Prakash, Siddheswar Maikap, Hsin-Ming Cheng, Ling-Na Tsai, Jian-Tai Qiu, Samit K. Ray

**Affiliations:** 1 Thin Film Nano Tech. Lab., Department of Electronics Engineering, Chang Gung University, 259 Wen-Hwa 1st Rd., Kwei-Shan, Tao-Yuan 33302 Taiwan; 2 Electronics and Optoelectronics Research Laboratories, Industrial Technology Research Institute (ITRI), Hsinchu, 310 Taiwan; 3 Division of Gyn-Oncology, Department of Obs/Gyn, Chang Gung Memorial Hospital (CGMH), Tao-Yuan, 33302 Taiwan; 4 Material and Chemical Research Laboratories, Industrial Technology Research Institute, Hsinchu, 310 Taiwan; 5 Department of Biomedical Sciences, School of Medicine, Chang Gung University (CGU), Tao-Yuan, 33302 Taiwan; 6 Department of Physics, Indian Institute of Technology, Kharagpur, 721302 India; 7 S. N. Bose National Centre for Basic Sciences, J D Block, Sector III, Salt Lake City, Kolkata, 106 India

## Abstract

Formation-free multi-level resistive switching characteristics by using 10 nm-thick polycrystalline GeO_x_ film in a simple W/GeO_x_/W structure and understanding of switching mechanism through redox reaction in H_2_O_2_/sarcosine sensing (or changing Ge°/Ge^4+^ oxidation states under external bias) have been reported for the first time. Oxidation states of Ge^0^/Ge^4+^ are confirmed by both XPS and H_2_O_2_ sensing of GeO_x_ membrane in electrolyte-insulator-semiconductor structure. Highly repeatable 1000 dc cycles and stable program/erase (P/E) endurance of >10^6^ cycles at a small pulse width of 100 ns are achieved at a low operation current of 0.1 µA. The thickness of GeO_x_ layer is found to be increased to 12.5 nm with the reduction of polycrystalline grain size of <7 nm after P/E of 10^6^ cycles, which is observed by high-resolution TEM. The switching mechanism is explored through redox reaction in GeO_x_ membrane by sensing 1 nM H_2_O_2_, which is owing to the change of oxidation states from Ge^0^ to Ge^4+^ because of the enhanced O^2−^ ions migration in memory device under external bias. In addition, sarcosine as a prostate cancer biomarker with low concentration of 50 pM to 10 µM is also detected.

## Introduction

Recently, resistive switching random access memory (RRAM) has been specified as one of the most progressive next generation nonvolatile memories to replace 3D flash due to its simple metal-insulator-metal structure, complementary metal-oxide-semiconductor (CMOS) compatibility, low power consumption, better uniformity, strong data retention, long endurance, and easier fabrication with low cost^1–4^. The RRAM devices with several high-κ materials such as Ta_2_O_5_^5, 6^, HfO_x_^7, 8^, TiO_2_^9^, BaTiO_3_^10^ have been reported by several groups. Along with these different oxides, the GeO_x_ material has profound potential to execute resistive switching owing to its compatibility with back-end-of-line (BOEL) process in CMOS technology, capability of producing oxygen vacancy at low temperature^11^, widely spanned dielectric constant values (k~12–15)^12^, large band gap (E_g_~4.3–5.9 eV)^13, 14^, and good thermal stability^15, 16^. There are only few studies on GeO_x_-based RRAM in literature. Applying plasma treatment, the current compliance (CC) is reduced to 600 µA in Ni/GeO_x_/TiO_y_/N^+^ TaN RRAM stack^17^. Bipolar switching behaviors using GeO_x_-based different memory structures have been demonstrated at CC of 3.5 µA^11^. A significant reduction of resistive switching variation has been achieved at a high CC of 50 mA in Au/Zr/GeO_x_/YSZ/TiN structure^18^. In addition, multi-level cell (MLC) operation is also very important for high-density data storage RRAM application. There are few reports on MLC operation by using Pt/Ta_2_O_5_/TiN^19^, Ta/TaO_x_/Pt^20^, TiN/HfO_x_/Pt^21^, Pt/HfO_2_/TiO_2_/ITO^22^ and TiN/Ti/TiO_2−x_/Pt NC/TiO_2−x_/Au^23^ structures. Further, the switching mechanism is not explored clearly. Apart from those different electrode materials, tungsten (W) as a electrode material is also very useful in metal-insulator-metal (MIM) structure because of its CMOS compatibility, easier to complete RESET process, high electron emissivity, thermally and chemically stable^24^. A simple formation-free W/GeO_x_/W RRAM device with low operation current of 0.1–100 µA and proper switching mechanism evaluation through H_2_O_2_ sensing at a low concentration of 1 nM by oxidation state changing from Ge (Ge^0^ to Ge^4+^) in electrolyte-insulator-semiconductor (EIS) structure has not been reported yet. Further, prostate cancer is most common male malignancy in the western world and accountable for second most common male cancer related deaths^25^. According to Koutros *et al*., sarcosine is one of the biomarkers of prostate cancer^26^. Therefore, sarcosine^27^ has been also detected by using electrolyte-insulator-semiconductor (EIS) structure in this study, which has been not reported yet.

In this study, we have manifested formation-free MLC resistive switching having the range of CCs from 0.1 µA to 100 µA and modulation of negative voltages by using GeO_x_ material in a simple W/GeO_x_/W structure and the switching mechanism through redox reaction in H_2_O_2_ sensing has been explored owing to oxidation states changing of Ge^0^/Ge^4+^ under external bias for the first time. High-resolution transmission electron microscope (HRTEM) image has confirmed the layer-by-layer structure of the 200 × 200 nm^2^ via-hole devices. The polycrystalline GeO_x_ material has Ge^0^ and Ge^4+^ oxidation states, which is confirmed by X-ray photo-electron spectroscopy. An excellent dc endurance of 1000 cycles and long P/E endurance of >10^6^ cycles with a small P/E pulse width of 100 ns can be achieved under a low operation current of 0.1 µA. The Fowler-Nordheim (F-N) tunneling conduction dominates at high field in HRS and low field regimes are complied with space-charge limited current conduction (SCLC). This structure has enhanced memory performances with good device-to-device switching uniformity, repeatable multi-level cell (MLC) by varying negative voltage with a high resistance ratio of 600, long P/E endurances of >10^6^ cycles at high V_read_ of −1V, and robust data retention of >10^5^s at 85 °C after 1000 P/E cycles. The HRTEM images at ‘SET’ condition of the device (after 10^6^ cycles of P/E) show the thickness increment of GeO_x_ layer (12.5 nm vs. 10 nm) along with small nanograins of <7 nm in diameter. The O^2−^ ions migration leads to conducting filament formation/rupture through redox reaction in GeO_x_ material and changes the Ge^0^/Ge^4+^ oxidation states. Therefore, this W/GeO_x_/W RRAM device has very high potential to become the successor of 3D flash non-volatile memory (NVM) in future. The GeO_x_ membrane in EIS structure detects a low concentration of 1 nM H_2_O_2_ through oxidation-reduction reaction in GeO_x_ material. Sarcosine as a prostate cancer biomarker has been also detected with a low concentration of 50 pM, which will be useful to diagnosis cancer patient at early stage in future.

## Results and Discussion

**Figure 1:**
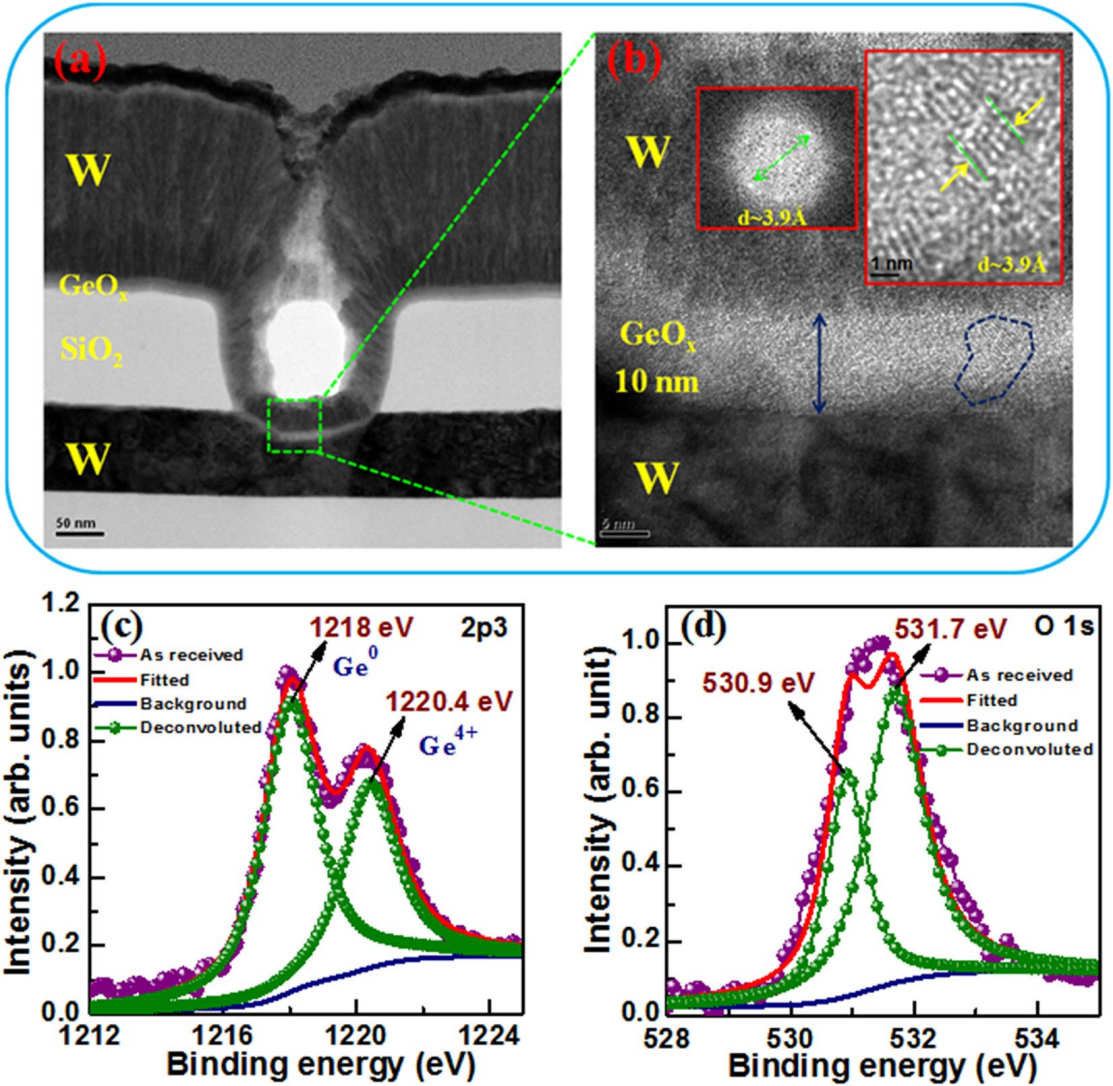
(**a**) TEM image of W/GeO_x_/W memory device having via-hole size of 0.2 × 0.2 µm^2^. (**b**) HRTEM image exhibits 10 nm-thick polycrystalline GeO_x_ film and corresponding FFT images (inset) exhibit the d-spacing value. XPS characteristics of (**c**) Ge*2p* and (**d**) O*1s* spectra for the GeO_x_ films on p-Si substrate.

Figure 1a reveals the cross-sectional TEM image with a small via-hole size of 0.2 × 0.2 µm^2^. Cross-sectional high-resolution TEM image inside the via-hole region quantifies the thickness of GeO_x_ switching material (SM) of approximately 10 nm (Fig. 1b). The Fast Fourier Transformation (FFT) images (inset) of marked region explore that the d-spacing value is 3.9 Å, which lies in between the d- spacing values of Ge (3.27 Å)^28^ and GeO_2_ (4.3 Å)^29^ nanocrystals. It assures the formation of polycrystalline GeO_x_ film with a grain size of approximately 10 nm in pristine device. In order to understand the elemental composition and defects in the GeO_x_ films, the chemical binding states have been investigated by XPS characterization (Fig. 1c,d). Figure 1c shows XPS spectrum of Ge*2p* core-level electrons, which is fitted by using Shirley background subtraction and Gaussian functions. The corresponding binding energies (BE) of the doublet de-convoluted Ge 2p_3/2_ spectrum peaks are centered at 1218 and 1220.4 eV. The lower BE peak (1218 eV) is related to the non-oxidized semiconductor Ge (Ge^0^), while the higher BE peak (1220.4 eV) is assigned to the Ge-O bonding i.e, oxidized Ge^4+^ state^30, 31^. Bodlaki *et al*.^30^ and Wu *et al*.^31^ have reported elemental Ge2p_3/2_ (Ge^0^ state) peak at 1217.9 and 1217.8 eV, while the Ge^4+^ 2p_3/2_ state exists at 1220.6 eV and 1220.1 eV, respectively. The binding energy peaks are very close to our present observation. The estimated ratio of Ge^0^/Ge^4+^ peak is approximately1.6. Therefore, the formation of GeO_x_ (x ≤ 2) film is confirmed. The O*1s* peak (BE of 530.9 eV) denotes the associated oxygen ions (O^2−^) in GeO_x_ matrix, as shown in Fig. 1d. Higher BE peak at 531.7 eV addresses to the defects i.e, oxygen deficient (V_O_) in GeO_x_ layer. Higher BE peak specifies the presence of large amount V_O_’s in GeO_x_ film, as shown in Fig. 1d. In similar way, it is reported that the position of main O*1s* peak which remains bonded with GeO_x_ at BE of 531.2 ± 0.2 eV while the rest one at 532.1 ± 0.2 eV indicates the chemisorbed oxygen^32^. In our study, V_O_’s plays the most important role to originate the formation-free resistive switching via oxidation state changes of Ge^4+^/Ge^0^ in W/GeO_x_/W structure. Yang *et al*.^33^ have also reported the O^2−^ ions migration and oxidation state changes from Mo^5+^ to Mo^6+^ in Ag/MoO_3−x_/FTO resistive switching memory under external bias. Multi-level resistive switching through H_2_O_2_ sensing mechanism in W/GeO_x_/W structure has been discussed later.

**Figure 2:**
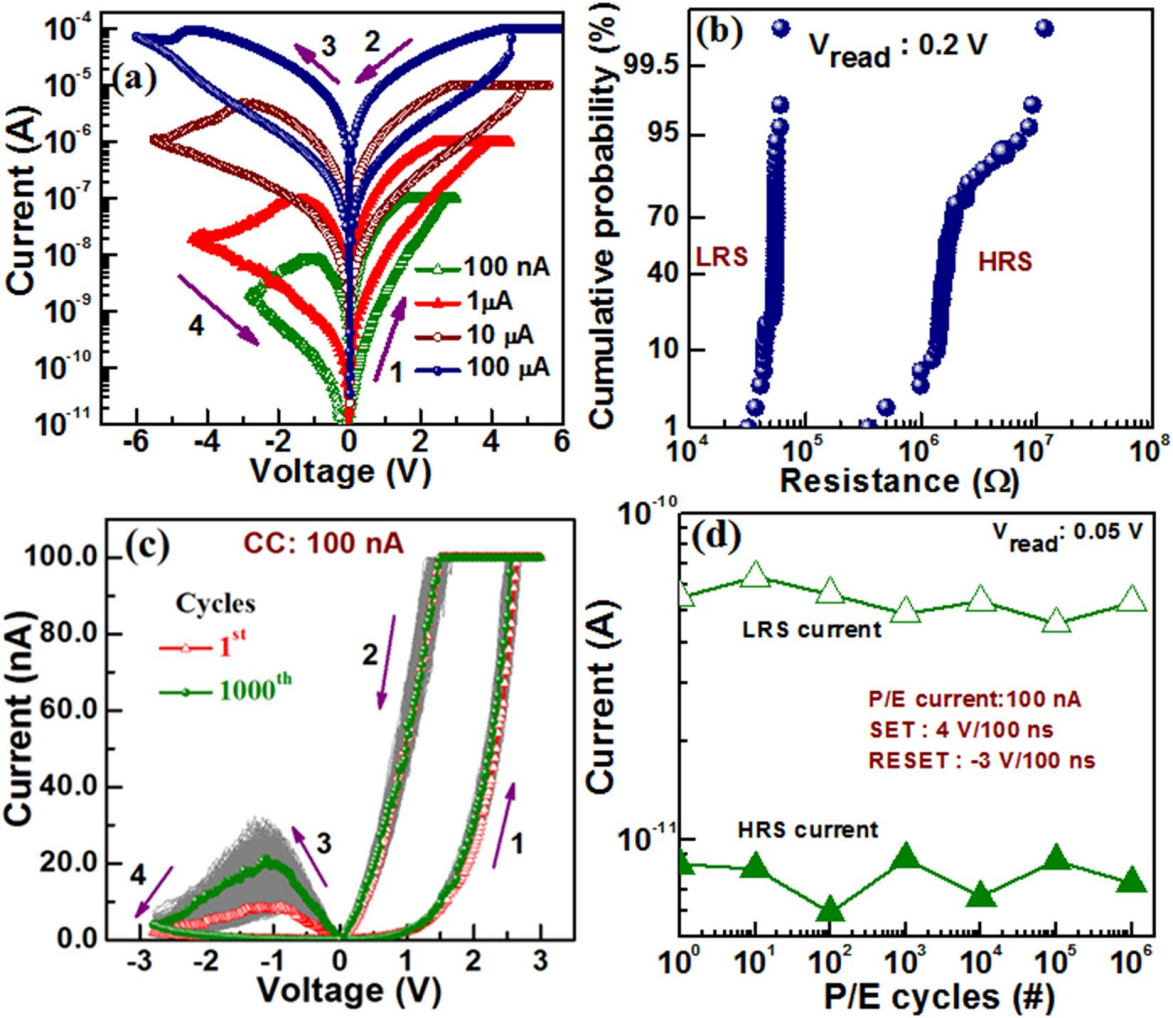
(**a**) Bipolar I-V resistive switching characteristics having CCs from 0.1 to 100 µA and (**b**) device-to-device cumulative probability plot at a CC of 100 µA. (**c**) Consecutive > 1000 dc I-V cycles. (**d**) The P/E endurance of >10^6^ cycles at a low operation current of 100 nA and a small P/E pulse width of 100 ns is applied.

Figure 2a shows the formation-free I–V hysteresis characteristics at current compliances of 0.1, 1, 10, and 100 µA. The voltage sweeping directions are indicated by the arrows: 1 → 2 → 3 → 4. By applying +Ve bias of +3 V on the top electrode (TE), the memory device switches from high resistance state (HRS) to low resistance state (LRS). The SET voltage (V_SET_) is approximately 2.6 V at a low CC of 100 nA. Initially no extra formation voltage process is needed, which is very necessary to reduce power consumption or reduce the process steps in memory circuit. On the other hand, the RRAM device starts to switch back to HRS at the V_RESET_/I_RESET_ of −0.9 V/8.8 nA. Therefore, this device can be operated with ultra low SET/RESET current of 0.1 µA/8.8 nA at V_SET_/V_RESET_ of +2.6 V/−0.9 V, which is very important for low energy memory device application. In similar way, the resistive switching occurs at the CCs of 1, 10 and 100 µA having V_SET_/V_RESET_ of +3.7/−1.3, +4.8/−3 and +4.5/−4.35 V, respectively. All resistance states at each CC can be repeated more than 1000 cycles following the same track (Supplementary Information; Fig. S1). Both HRS and LRS are decreasing with increasing CCs, which can be controlled by controlling CCs. This suggests that the oxidation-reduction occurs at the W TE/GeO_x_ interface or interface-type^34^ resistive switching. The HRS current can be decreased with increasing V_STOP_, which has been explained later. Good switching reliability is one of the challenging issues to implement RRAM for real application. We have analyzed cumulative probability distribution of randomly chosen 50 devices at a CC of 100 µA to confirm the reliability (Fig. 2b). Average values (σ_m_)/standard deviation (σ_s_) for LRS and HRS are found to be 52.5 kΩ/5.7 kΩ and 2.43 MΩ/2.18 MΩ, respectively at a read voltage (V_read_) of 0.2 V. Hence the dispersion (σ_s_/σ_m_) values are very small of 0.1 and 0.9 for LRS and HRS, respectively. Therefore, the device-to-device switching uniformity yield is good (96%) with higher resistance ratio (HRS/LRS) of approximately 46. To ensure the low current operation reproducibility, Fig. 2c exhibits the resistive switching characteristics of 1000 successive dc switching cycles at 0.1 µA. It is noticed that this structure can produce uniform switching with acceptable resistance ratio of approximately 15 even after 1000 dc cycles, which has been presented also in the Supplementary Information (Fig. S2). In addition, this device shows stable operation with more than 10^6^ P/E cycles under a low P/E current of 100 nA and a small P/E pulse width of 100 ns (Fig. 2d). This implies that the GeO_x_ SM in a simple W/GeO_x_/W structure plays a major role to achieve such a low current operation.

**Figure 3:**
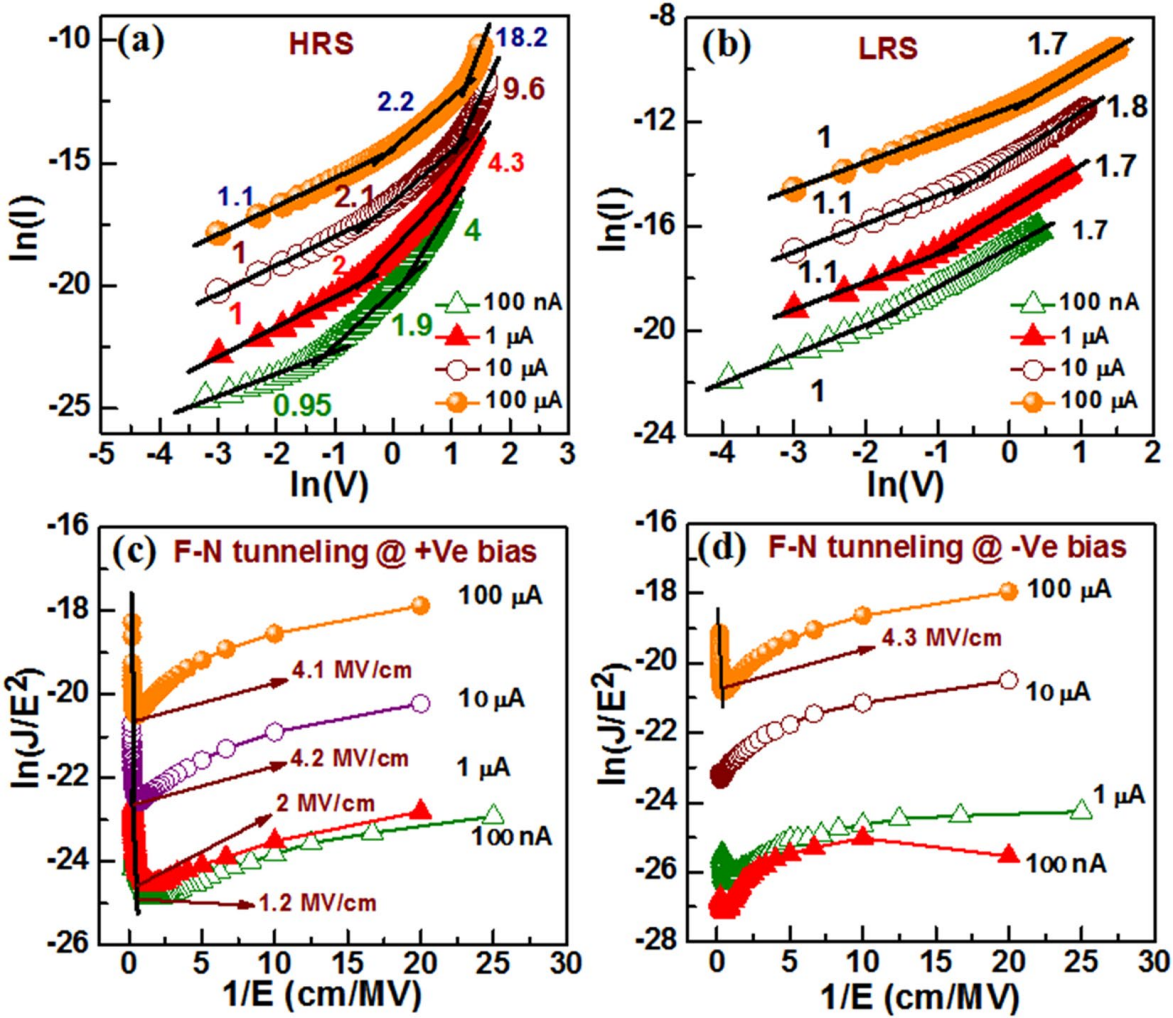
Linear fitting of I-V curves in log-log scale at (**a**) HRS and (**b**) LRS current show SCLC nature. F-N tunneling conduction occurs at E_C_ > 3 MV/cm (**c**) in +Ve bias and (**d**) −Ve bias of the HRS currents. The E_C_ is critical electric field. The F-N tunneling is observed at higher CC of >10 µA.

To understand the current transport mechanism, double log scale fitting of I-V curves at room temperature in both HRS (Fig. 3a) and LRS (Fig. 3b) is analyzed. There are three regions in HRS current having slope values vary from (i) 0.95 to 1.1 at low bias region then (ii) 1.9 to 2.3 at middle bias region, and finally those values are varied from (iii) 4 to 18.2. On the other side, LRS current consists of two regions having (i) slope values vary from 1 to 1.1 at low bias region and then (ii) 1.7 to 1.8 at higher bias region. Both HRS and LRS currents follow linear relationship, i.e, IαV at low voltage and then quadratic relationship (i.e, IαV^2^) at high voltage. It signifies that both HRS and LRS currents are Ohmic where thermally generated free electron density becomes much more than the injected electron density from the W electrode. By increasing bias the injected carrier density increases immensely than the thermally generated free carrier density which results current increment rapidly. Thus HRS currents are complied with trap-charge controlled SCLC mechanism. The voltage at which current increases rapidly is reported trap–filled-limited voltage (V_TFL_). Corresponding trap density (n_t_) has been calculated using the following equation^35^,

1 $${\eta }_{t}=\frac{2\varepsilon {\varepsilon }_{0}{V}_{TFL}}{q{d}^{2}}$$

where *n*_*t*_ is trap density, *q* is the electronic charge, ε (~12) is the dielectric constant of GeO_x_, *ε*_0_ is free-space permittivity, *d* (~10 nm) is the thickness of switching layer. The V_TFL_ values of HRS current at CCs of 0.1, 1, 10 and 100 µA are found to be 1.2, 2.3, 2.9, and 3.3 V, while the *n*_*t*_ values are found to be 1.59 × 10^19^, 3.05 × 10^19^, 3.85 × 10^19^ and 4.4 × 10^19^ cm^−3^, respectively. Similarly, the V_TFL_/n_t_ values of 2.48 V/6.67 × 10^16^ cm^−3^ and 0.46 V/1.22 × 10^16^ cm^−3^ are reported for FTO/TiO_2_ and TiO_2_/CH_3_NH_3_PbI_3_/Ag structure, respectively^35^. Higher slope values of HRS current are 9.6 and 18.2 at CC of 10 and 100 µA, respectively, which are owing to F-N tunneling phenomena. Kim *et al*. have reported analogous F-N tunneling phenomena at higher slope value of 6.6 for the HRS currents above V_TFL_ and SCLC mechanism is reported at LRS current in their ITO/GaZnO/ITO structure^36^. To ensure the F-N tunneling conduction, experimental I-V curves of the HRS currents in both + Ve and –Ve bias regions are plotted as ln (J/E^2^) vs. 1/E (Fig. 3c,d), where *E* is the electric field. The tunneling barrier heights (Φ_b_) have been calculated from the ln (J/E^2^) vs. 1/E plots using the following F-N tunneling equation below^37^,

2 $${{\rm{\Phi }}}_{b}={(-S)}^{\frac{2}{3}}{\{\frac{{(\frac{3}{4}q\hslash )}^{2}}{2{m}_{ox}}\}}^{\frac{2}{3}}$$

where S is the slope, *m*_*ox*_ is the tunneling effective mass of electron (or holes) in the GeO_x_ layer and $\hbar$ is the reduced Planck’s constant. The linear fitting nature at CCs of 10 and 100 µA confirms the F-N tunneling conduction above critical electric field (E_C_) of ≥3 MV/cm^38^. Considering *m*_*ox*_ = *0.2m*_0_^39^, the calculated values of Φ_b_ are 1.14 eV and 0.71 eV at CC of 100 µA for +Ve and -Ve biases, respectively. The Φ_b_ values at the TE/GeO_x_ interface is lower (0.71 eV vs. 1.14 eV) because of more defective than the BE/GeO_x_ interface. Similarly, the Φ_b_ value is 1.32 eV at +Ve bias for CC of 10 µA. Therefore, electrons tunnel through the triangular potential barrier at W/GeO_x_ interface into the conduction band of GeO_x_ film. Since F-N tunneling phenomena have been addressed. On the other hand, the F-N tunneling phenomena are eliminated because of lower bias. Therefore, this is suggested that the HRS current at higher bias of ≥3 V is dominated by F-N tunneling.

**Figure 4:**
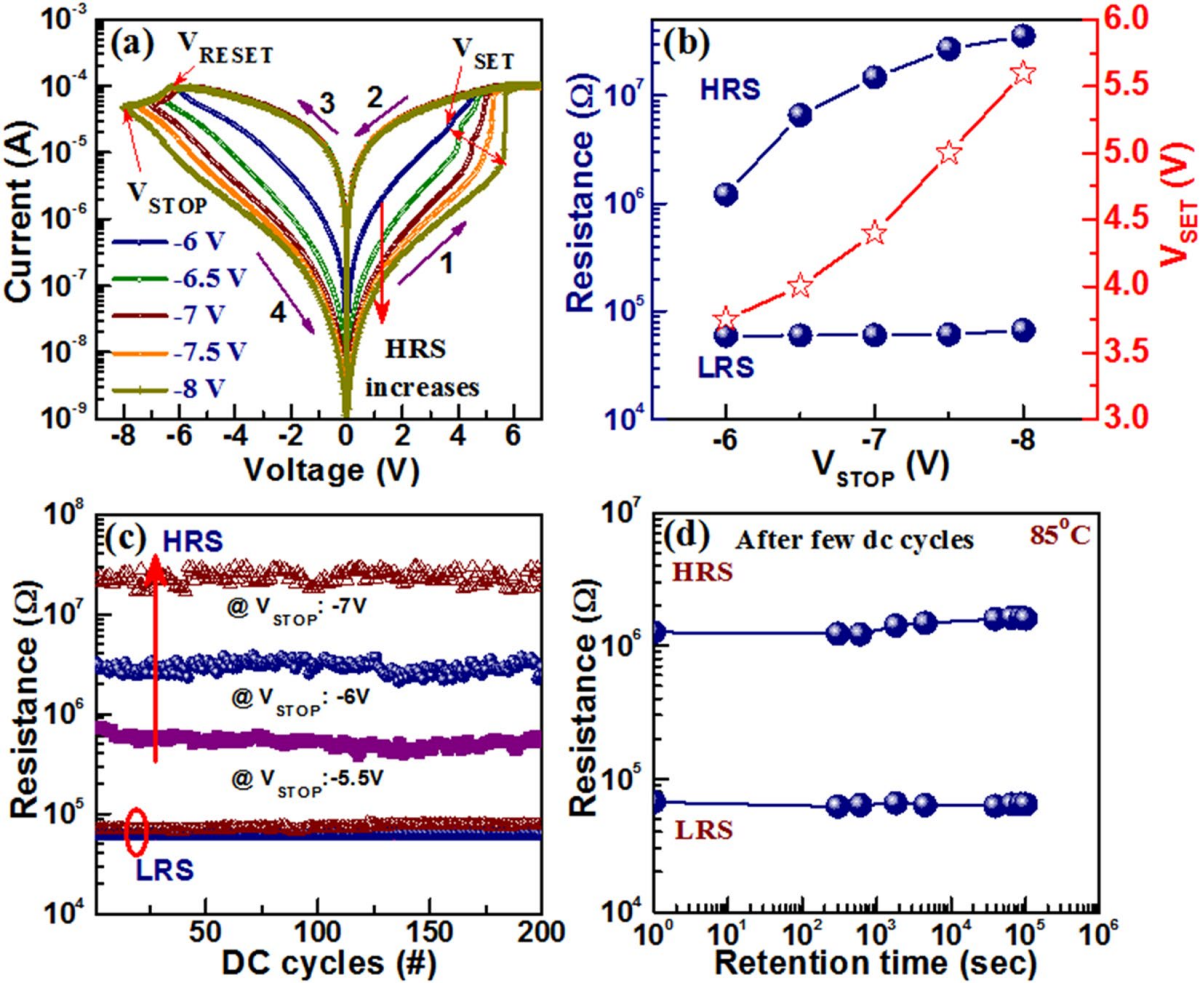
(**a**) Influence of V_STOP_’s on HRS currents, (**b**) Both HRS and V_SET_ increases with increasing V_STOP_ values while LRS value is independent. (**c**) MLC operation of HRS with dc cycles by varying V_STOP_ values. (**d**) Long data retention of >10^5^ sec at 85 °C is obtained after 1000 dc cycles.

Figure 4a shows the typical MLC operation by varying V_STOP_ voltages from −6 to −8V at a CC of 100 µA. The LRS value is independent of the V_STOP_. However, both resistance ratio and V_SET_ increase with increasing the V_STOP_ values (Fig. 4b). This implies that the oxidizing length of the conducting filaments is increased with increasing the V_STOP_ values. The corresponding Φ_b_ values also increases from 0.61 eV to 1.12 eV with increasing V_STOP_ value from −6 V to −8V, which is owing to gradual generation of Ge^4+^ ions (explained later). The resistance ratio becomes approximately 600 at a V_STOP_ of −8 V. Stable MLC operation of successive 200 dc cycles at different V_STOP_ values are shown in Fig. 4c. Hseih *et al*.^40^ have also executed similar MLC by varying V_STOP_ at higher CC of 1 mA in Ti/MgZnO/Pt structure. Therefore, it can be asserted that the W/GeO_x_/W structure is worthy of low current multilevel RRAM application under modulation of RESET voltages. From Fig. 4a, the HRS current decreases with increasing V_STOP_ and the device can be operated at approximately 5 µA after V_STOP_ of −8 V. From Fig. 2(a), the HRS currents are not overlapped owing to pristine defects related mechanism at low current operation of <1 µA. As long as the device is operated at higher current (>1 µA), it may not be easy to operate at 100 nA further or highly oxygen reservoir is needed at the W TE/GeO_x_ interface to re-oxidize the defects. On the other hand, the device should be operated at low current of <1 µA only. However, further study is needed to operate the device at 100 nA by increasing V_STOP_ voltage. This memory device can also perform robust data retention at 85 °C, as shown in Fig. 4d. The data retention is measured after 1000 dc cycles. Both LRS and HRS show long data retention time of >10^5^ s without any degradation. In order to describe the bipolar resistive switching process, V_O_ based filamentary-type conduction concept has been established in W/GeO_x_/W RRAM structure. At first +Ve bias is applied on TE and generated O^2−^ ions are driven towards TE leaving behind the V_O_’s. These O^2−^ ions are congregated at the TE/GeO_x_ interface having less possibility of WO_x_ layer formation because Gibbs free energy of GeO_2_, WO_3_ and WO_2_ are very close to each other say, −518.5, −510 and −506 kJ/mole at 300 K, respectively^11, 24, 41^. Thus gradually trap density at the TE/GeO_x_ interface gets lowered and triangular potential barrier is formed which is tunneled by electrons (e^−^) at the higher electric field. As a result, the TE and BE are connected by V_O_’s-based filamentary path through which electronic charges flow or the Ge^0^ based filament is formed under SET operation and the device turns into LRS. Due to semiconductor in nature of Ge^0^ (or Ge^1+^, Ge^2+^, Ge^3+^), the SCLC conduction is observed in LRS current (Fig. 3a,b). On the reverse bias, the filament is dissolved by the recombination with repelled O^2−^ ions and a dissolution gap is generated by increasing higher oxidation states or Ge^0^ to Ge^4+^ states. Eventually device switches back to HRS. Multi-level HRS is observed owing to more generation of Ge^4+^ oxidation states under RESET, which is also explained through H_2_O_2_ sensing later.

**Figure 5:**
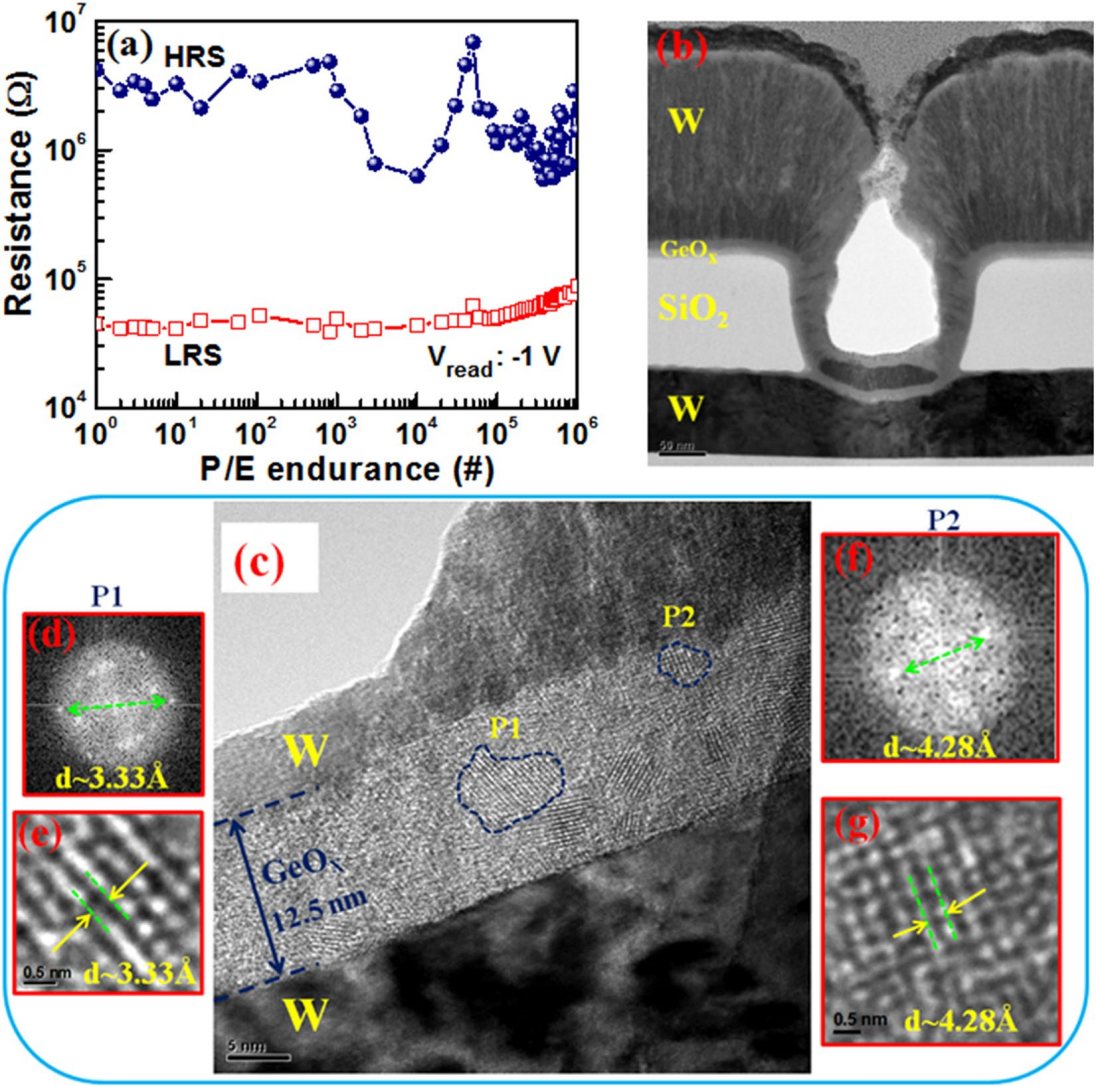
(**a**) P/E endurance of >10^6^ cycles at high V_read_ of −1 V. (**b**) TEM and (**c**) HRTEM images are obtained after P/E endurance of 10^6^ cycles which shows the increment of thickness of GeO_x_ switching layer than the pristine one (12.5 nm vs. 10 nm). Corresponding FFT images show the formation of (**d,e**) Ge and (**f,g**) GeO_2_ nanograins or nanocrystals with a small size of 2–7 nm in diameter.

To investigate the resistive switching mechanism inside of the polycrystalline GeO_x_ layer, the TEM images have been taken at ‘SET’ condition of the memory device after a long P/E endurance of 10^6^ cycles at a high V_read_ of −1V without any series transistor (Fig. 5a). This long endurance is obtained due to our novel W/GeO_x_/W structure design. The P/E current, voltage, and pulse width are 100 µA, +6/−6.5 V, and 0.5 ms, respectively. A little variation of HRS is observed than the LRS value because no resistance verification circuit is used. However, the large V_read_ of −1V is used to read during P/E cycles which will be easier for circuit design. There is no report of the switching material and investigation of mechanism after randomly 10^6^ P/E cycles operation, as we have reported in Fig. 5b,c. Basically, a structural change is observed due to combined effect of O^2−^ ions movement and thermal agitation, as shown in Fig. 5c (inset). Isolated small nanograins or nanocrystals (pointed by P1 and P2) with diameters of approximately 2 to 7 nm are observed clearly (density of 2 × 10^12^/cm^2^) at ‘SET’ state. The FFT image (Fig. 5d) and plane spacing image (Fig. 5e) of Ge nanograins show that the d-spacing value is 3.33 Å at the point P1. This is alike to the reported d-spacing values of 3.27 Å and 3.3 Å for Ge nanocrystal in (111) plane^28, 42^. Along with that, the d-spacing value of 4.28 Å is obtained from the FFT images (Fig. 5f,g) at the point P2 which indicates the formation of hexagonal GeO_2_ quartz like nanocrystals at the TE/GeO_x_ interface. Liu *et al*. have demonstrated the formation of hexagonal quartz like GeO_2_ nanocrystals with the d-spacing value of 4.3 Å in (01$\bar{1}$0) plane^29^. Those d-spacing values are quite different as compared to the as-deposited polycrystalline GeO_x_ SM at pristine state (d = 3.9 Å), as shown in Fig. 1b. Even the grain size is very small after 10^6^ P/E cycles as compared to pristine one (<5 nm vs. 10 nm). This is due to migration of O^2−^ ions under electrical thrust during SET operation towards the TE/GeO_x_ interface. Consequently, thickness augmentation of the GeO_x_ layer is also observed (Fig. 5c) at the SET state than the pristine state (~12.5 nm vs. 10 nm). In addition, sufficient localize joule heating factor during random P/E operation also trigger to construct nanograins in the GeO_x_ layer. Qian *et al*.^43^ have shown crystallization from TEM images due to joule heating effect in ITO/WO_3_/ITO RRAM structure. To understand the switching mechanism, further study has been explained below.

**Figure 6:**
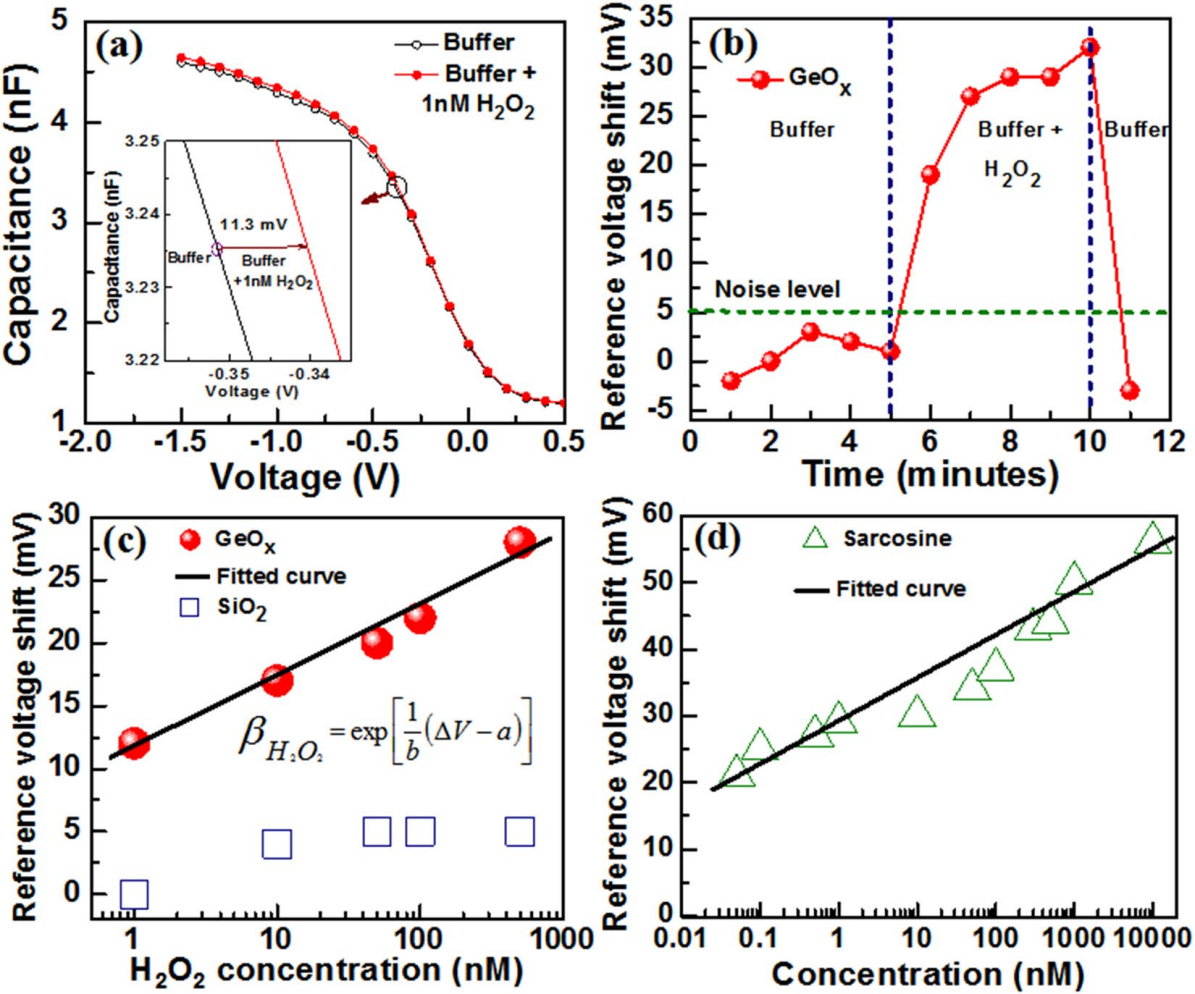
(**a**) C-V curve by adding 1 nM H_2_O_2_ in pH7. The reference voltage shift is 11.3 mV, as shown in inset. (**b**) Time-dependent reference voltage shift with and without H_2_O_2_ in PBS buffer solution. This sensor shows reversible phenomena because the GeO_x_ sensing membrane has redox characteristics. It implies that the sensor can be re-used. This reversible phenomenon makes us understand the resistive switching mechanism through changing Ge^0^/Ge^4+^ oxidation states. (**c**) Comparison of H_2_O_2_ detection characteristics between GeO_x_ and bare SiO_2_ sensing membranes. The GeO_x_ membrane can sense H_2_O_2_ with concentration ranging from 1 nM to 500 nM, which is good for real application. (**d**) The reference voltage shift versus sarcosine concentration from 50 pM to 10 µM.

**Figure 7:**
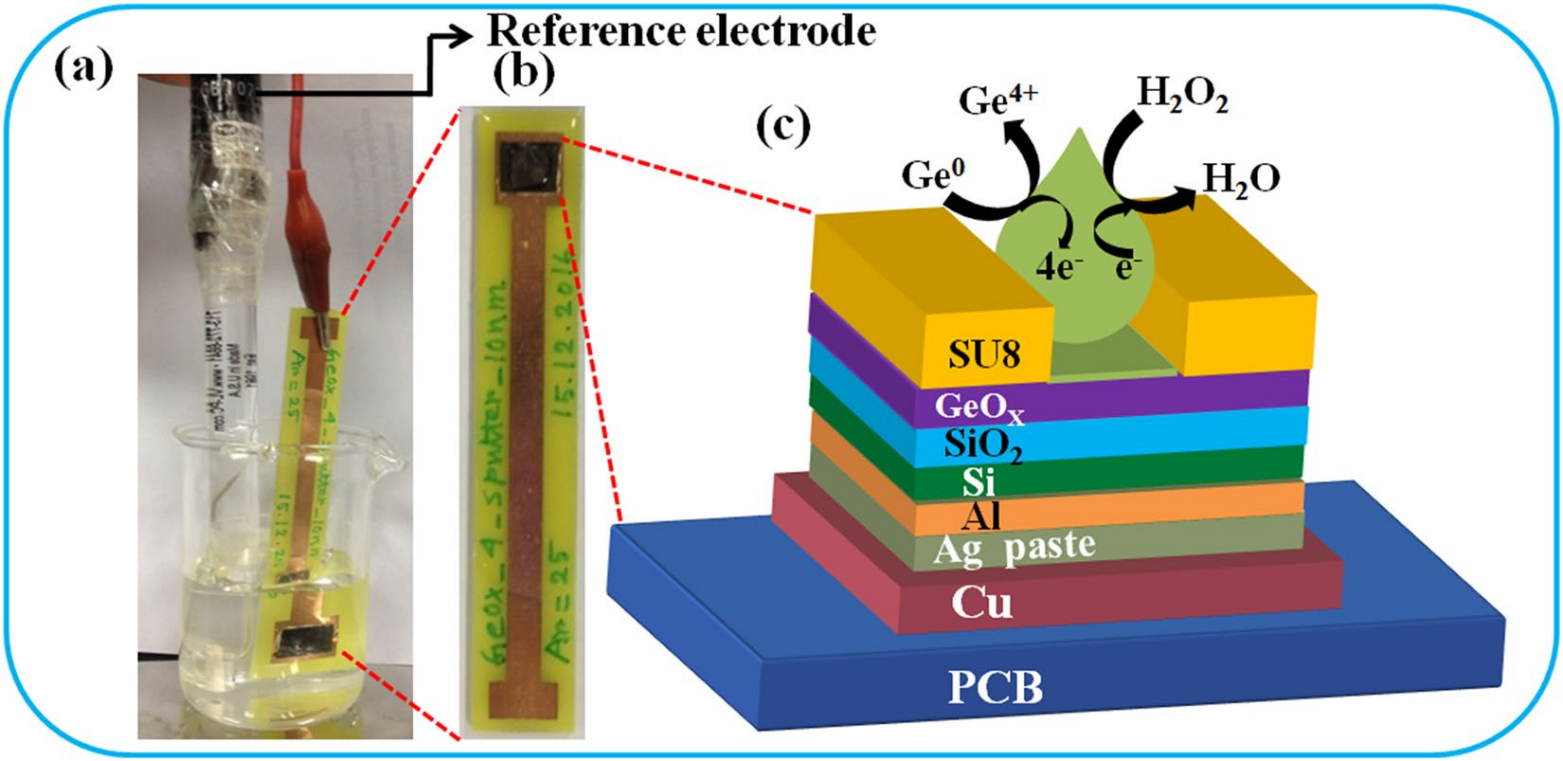
(**a**) The sensor chip and Ag/AgCl reference electrode are immersed in PBS buffer solution. (**b**) Sensor chip mounted on PCB and epoxy was used to isolate in between Cu line and sensor. (**c**) Schematic diagram of GeO_x_-based sensor in electrolyte-insulator-semiconductor structure and oxidation-reduction is shown. This measurement system sets up in our lab.

Figure 6 shows the H_2_O_2_ and sarcosine sensing by using GeO_x_ membrane in EIS structure. Figure 7 shows measurement set up for GeO_x_-based sensing membrane in EIS structure. The as-deposited GeO_x_ membrane shows the pH sensitivity of approximately 33 mV/pH with excellent linearity of 99.8% (Supplementary Information; Fig. S3). Adding different pH buffer solutions, the surface charge on membrane is changing. By increasing pH value, the OH^−^ ions are adsorbed on the sensing surface or reduction of H^+^ ions. On the other hand, de-protonation^44^ occurs on the sensing membrane surface as well as amount of surface potential is reduced. The Si band bending is also decreased as compare to lower pH value. This needs less potential to have a flat-band condition. As a result, the flat-band voltage or reference voltage is shifted towards positive direction. Therefore, the protonation/de-protonation (i.e., H^+^/OH^−^) of the sensing membrane in contact of different pH values changes the reference voltage, which is measured by C-V curves. A similar pH sensitivity of approximately 35 mV/pH is observed for bare SiO_2_ membrane^45^. In addition, the H_2_O_2_ is an active oxidizing agent which has more application in bio-medical research and will be used here to identify the oxidation states as well as the switching mechanism has been investigated. Figure 6a shows the reference voltage shift with a low concentration of 1 nM H_2_O_2_. The reference voltage shift is approximately 11 mV in presence H_2_O_2_ with PBS (pH 7) solution (inset of Fig. 6a). Our low concentration H_2_O_2_ sensing (1 nM) is comparable with other reported values of 5 nM–3.1 µM by cyclic voltametry measurement^46–50^. Voltage shift occurs due to oxidation of GeO_x_ membrane using following oxidation-reduction equation below.

3 $$Ge\leftrightarrow G{e}^{2+}+2{e}^{-}\leftrightarrow G{e}^{4+}+4{e}^{-}$$

From equation (3), the oxidation state of Ge changes from Ge^0^ to Ge^4+^ via Ge^1+^, Ge^2+^, Ge^3+^ states^51^. First, H_2_O_2_ in PBS buffer solution will receive electron (e−) from the GeO_x_ surface (Fig. 7e). This will produce OH^−^ ions and OH^*^ radicals in solution (H_2_O_2+_e^−^ ↔ OH^− + ^OH^*^; OH^*^ + e^−^ ↔ OH^−^). Finally, the OH^−^ ions will receive H^+^ ions from the PBS buffer solution and will produce H_2_O. In this exchange reaction, the pH value of 7 will be unchanged. Basically, the GeO_x_ membrane will be oxidized and the reference voltage will be changed. The work function of Ge is 4.67 eV^52^ and it is increased to 4.94 eV^53^ for GeO_2_. By considering the doping (5 × 10^15^ cm^−3^) of p-Si, the work function is 4.94 eV. Therefore, the work function of GeO_x_ membrane increases with increasing H_2_O_2_ concentration as well as Si band bending is reduced. As a result, the reference voltage is shifted towards positive direction. Previously, we have also reported the oxidation states changed of Zn to ZnO in contact of H_2_O_2_^45^. Figure 6b shows time-dependent H_2_O_2_ response with a concentration of 500 nM. The C-V curve is measured at a regular time interval of 1 minute and the reference voltage is plotted with time. It is clear that the GeO_x_-based sensor retains to its original response in PBS buffer solution after 5 minutes of H_2_O_2_ sensing. Therefore, this sensor can be used again or the H_2_O_2_ sensing is reversible. Similarly, the resistive switching is also reversible under SET and RESET operation because of redox reaction in GeO_x_ material. The reference voltage shift gradually increases with successive addition of H_2_O_2_ from 1 nM to 500 nM, where as bare SiO_2_ based membrane is unable to execute any voltage shift (Fig. 6c). Therefore, the GeO_x_ membrane has redox properties. We have fitted the reference voltage shift curve of GeO_x_ sensing membrane and generated mathematical equation is below,

4 $${\beta }_{{H}_{2}{O}_{2}}=\exp [\frac{1}{b}({\rm{\Delta }}V-a)]$$

where, *β*_*H2O2*_ is the concentration of H_2_O_2_, ∆V is the corresponding reference voltage shift, ‘b’ and ‘a’ are the slope and intercept of the fitted curve having valued 5.7 and 11.4, respectively. From the above equation (4), a required H_2_O_2_ concentration can be calculated at any arbitrary voltage shift. Figure 6d shows the reference voltage shift with increasing sarcosine concentration from 50 pM to 10 µM. Sarcosine prostate cancer biomarker reacts with enzyme sarcosine oxidase and produces H_2_O_2_ as shown equation (5) below.

5 $$Sar\,\cos \,ine+{H}_{2}O+{O}_{2}\mathop{\longrightarrow }\limits^{\begin{array}{c}Sar\,\cos \,ine\\ oxidase\end{array}}Glycine+HCHO+{H}_{2}{O}_{2}$$

From fitting curve, the values of ‘b’ and ‘a’ are found to be 6.1 and 28, respectively. A minimum concentration of 50 pM sarcosine is detected. Both H_2_O_2_ and sarcosine have similar relationship. The pH7 value does not change during successive addition of sarcosine in pBS buffer with enzyme. On the other hand, pure sarcosine with PBS buffer or enzyme with PBS buffer solution does not show reasonable sensing. Therefore, sarcosine concentration (enzyme with PBS buffer solution) will be detected from the reference voltage shift as well as the sensor will be useful to detect early stage of prostate cancer patient in future or prostate cancer can be monitored. Basically, the H_2_O_2_/sarcosine sensing by using GeO_x_ membrane or switching material proves that the O^2−^ ions migration lead to the oxidation-reduction through changing of Ge^0^/Ge^4+^ oxidation states under external bias. This is responsible the switching mechanism and the multi-level occurs owing to more generation of Ge^0^ under SET or Ge^4+^ under RESET. Therefore, the GeO_x_ material is not only attractive for multi-level resistive switching memory in a simple W/GeO_x_/W structure but also could be potentially useful for H_2_O_2_/prostate cancer detection in near future.

## Conclusion

In conclusion, forming-free MLC bipolar resistive switching characteristics and switching mechanism using a simple W/GeO_x_/W structure, and evidence of redox reaction in GeO_x_ material through H_2_O_2_ sensing by changing oxidation state of Ge^0^/Ge^4+^ have been reported for the first time. The oxidation states are confirmed by XPS. Via-hole device with a size of 200 × 200 nm^2^ and polycrystalline grain in GeO_x_ material are investigated by HRTEM. The W/GeO_x_/W memory device is able to execute a stable dc endurance of 1000 cycles at ultra low CC of 0.1 µA. The SCLC conduction leads in LRS and low field regime of HRS. But, F-N tunneling dominates in high field regime (>3MV/cm) of HRS. This stack also produces good uniformity, long P/E endurances of >10^6^ at high V_read_ of −1V, and excellent data retention of >10^5^ s at 85 °C after few dc cycles at a low operation current of 100 µA. The HRTEM at SET state of the device (after 10^6^ P/E cycles) shows the Ge and GeO_2_ nanograins with small size of <7 nm as well as the thickness is expanded than the pristine one (12.5 nm vs. 10 nm) due to O^2−^ ions movement and joule heating effect or the conducting filament formation is due to O^2−^ ions migration towards TE/GeO_x_ interface as well as Ge^0^ formation through reduction of GeO_x_. Generation of Ge^0^ by increasing CC and generation of Ge^4+^ ions (via Ge^1+^, Ge^2+^, Ge^3+^) by increasing V_STOP_ values as well as barrier heights controls the LRS and HRS currents. The redox reaction and understanding of switching mechanism in GeO_x_ material confirm by measuring H_2_O_2_ sensing with concentration ranging from 1 nM to 500 nM in PBS buffer solution. In addition, sarcosine prostate cancer biomarker with a low concentration of 50 pM is detected successfully. Thus eventually it can be concluded that GeO_x_-based W/GeO_x_/W memory device as well as H_2_O_2_/sarcosine sensing will be very propitious for the forthcoming RRAM and bio-sensor for diagnosis of human diseases as well as early detection of prostate cancer in near future.

## Methods

### Memory device fabrication

Resistive switching memory device with a small via-hole size of 0.2 × 0.2 µm^2^ was fabricated on 200 nm-thick SiO_2_ layer. The 8-inch p-type Si wafers were used. A 100 nm-thick W bottom electrode (BE) was deposited by RF magnetron sputtering. To define an active area, a 150 nm-thick SiO_2_ layer was deposited on the BE. Standard lithography and etching processes were used to expose the active area. Photo-resist was used to open active area and the top electrode (TE) regions. Then, a GeO_x_ switching material (SM) with a thickness of approximately 10 nm was deposited by RF sputtering process by using Ge target (purity: 99.99%) at room temperature. The base pressure of the chamber was 2 × 10^−5^ Torr. Argon (Ar) gas with a flow rate of 25 sccm had been kept fix during deposition and the deposition power was 50 W. The residual oxygen (O_2_), inside the chamber had been deliberately allowed to react during the deposition of Ge, which created GeO_x_ layer. Then, a W top electrode (TE) with a thickness of approximately 200 nm was deposited by the same RF sputtering process using W target (purity: 99.9%). Ar flow rate was 25 sccm and deposition power was 150 W. The deposition time was 30 min. Finally, lift-off process was done to get W/GeO_x_/W RRAM device with a size of 0.2 × 0.2 µm^2^. The thickness and microscopic structure of GeO_x_ layer in W/GeO_x_/W RRAM device were analyzed by transmission electron microscopy (TEM-JEOL 2100F) with 200 keV energy. Deposition repeatability had been checked by preparing samples three times under invariant conditions along with their electrical measurements. All the electrical characteristics have been measured by Agilent 4156C precision semiconductor analyzer in our lab. Throughout the measurement, sweeping bias is applied on the TE, keeping BE as grounded.

### Sensor fabrication

A 4-inch p-type Si wafer was cleaned by standard Radio Corporation of America (RCA) process. Then, a 40 nm-thick SiO_2_ layer was grown on Si by dry oxidation process with a temperature 950 °C in presence of 2.5 sccm O_2_ gas flow for 70 min. Then, a 10 nm-thick GeO_x_ film using Ge target was deposited by RF sputtering process. Back side SiO_2_ layer from the wafers was removed by buffer oxide etchant (BOE) solution. Then, a 300 nm-thick Al back contact was grown by thermal evaporation process. The sensing area of 3.24 mm² was defined by SU-8 negative photo resist using photo-lithography technique. Finally, the sensor was placed on copper coated printed circuit board (PCB) through Ag paste and it was encapsulated by epoxy to isolate solution from the Si and PCB. Both PCB chip and an Ag/AgCl reference electrode were placed inside a beaker. A 5 ml solution kept in a small glass beaker. To reduce outside noise during capacitance-voltage (C-V) measurement, the glass beaker kept inside black box, which one was purchased from Super Solutions & Services Co., Ltd, Hsinchu, Taiwan. The sensor and reference electrode were connected to HP 4284 A *LCR* semiconductor precision analyzer with computer interfacing. The measurement was controlled the computer and collected all data. The reference electrode and PCB chip are shown in Fig. 7a. The optical microscope image of the PCB chip is shown in Fig. 7b. A schematic view of the sensor in electrolyte-insulator-semiconductor (EIS) structure is shown in Fig. 7c. The oxidation mechanism in contact of H_2_O_2_ solution on GeO_x_ surface is also demonstrated.

### pH, H_2_O_2_ and sarcosine solution preparation

In order to check the pH sensitivity and H_2_O_2_ sensing in EIS structure, the pH solutions from 2 to 10 were prepared in the following process. At first, 30 ml of each pH2 to pH10 solution was taken directly into different containers. All the pH solutions were bought from Alfa Aesar Company. Then, the Ag/AgCl reference electrode along with the GeO_x_ based sensor had been dipped into each solution. The corresponding C-V characteristics were measured at 100 Hz frequency by applying electrode bias in the voltage range from −1.5 volt to 0.5 volt. By calculating the reference voltage shifts at 60% maximum capacitance from pH2 to pH10, the pH sensitivity value was calculated accordingly. For H_2_O_2_ measurement firstly a 1 µM H_2_O_2_ stock was prepared by diluting 10 M H_2_O_2_ concentration with DI water. Later on the PBS (Phosphate Buffer Silane) solution has been prepared by mixing sodium phosphate (Na_2_HPO_4_), sodium diphosphate (NaH_2_PO_4_), sodium chloride (NaCl) and DI water in proper way. The mentioned solutions were purchased from J. T. Baker, Avantor Performance Materials Incorporation. Then, the C-V characteristics are measured by taking 5 ml of 5 mM pH7 PBS solution in a beaker. Then, the reference voltage shifts at 60% of maximum capacitance were measured and the reference voltage versus H_2_O_2_ concentration from 1 nM to 500 nM in PBS solution was plotted.

First, 100 ml of TRIS buffer (purchased from UniRegion Bio-Tech) was diluted in DI water and the pH value was adjusted to pH 7 by monitoring pH meter. Then a stock solution of sarcosine-oxidase with 300 units was diluted in 500 µl TRIS. From the enzyme stock solution, 25 units of enzyme was added in 5 ml of pH7 PBS buffer solution. Sarcosine oxidase was purchased from TOYOBO CO. LTD, Osaka, Japan and sarcosine was purchased from Sigma-Aldrich, China. Now, a 10 mM of sarcosine stock was prepared in DI water. By diluting this stock solution, variation of sarcosine concentration ranging from 50 pM to 10 µM was successively added in the pH7 PBS buffer-enzyme solution and corresponding reference voltage shifts were plotted.

## Electronic supplementary material

Supplementary Information
