# Supplementary material for: Understanding of multi-level resistive switching mechanism in GeOx through redox reaction in H2O2/sarcosine prostate cancer biomarker detection
Source: Sci Rep. 2017 Sep 11;7:11240. doi: 10.1038/s41598-017-11657-4 (PMC5593955; doi:10.1038/s41598-017-11657-4)
Supplement: Supplementary file 1 — Supplementary Information [file 41598_2017_11657_MOESM1_ESM.pdf]

Supplementary Information

Understanding of multi-level resistive switching mechanism in GeO_x through redox reaction in H_2O_2/sarcosine prostate cancer biomarker detection

Subhranu Samanta[a], Sheikh Ziaur Rahaman[a,b], Anisha Roy[a], Surajit Jana[a], Somsubhra Chakrabarti[a], Rajeswar Panja[a], Sourav Roy[a], Mrinmoy Dutta[a], Sreekanth Ginnaram[a], Amit Prakash[a], Siddheswar Maikap[*,a,c], Hsin-Ming Cheng[d], Ling-Na Tsai[d], Jian-Tai Qiu[c,e] and Samit K. Ray[f,g]

[a]Thin Film Nano Tech. Lab., Department of Electronics Engineering, Chang Gung University, 259 Wen-Hwa 1st Rd., Kwei-Shan, Tao-Yuan, 33302, Taiwan

[b]Electronics and Optoelectronics Research Laboratories, Industrial Technology Research Institute (ITRI), Hsinchu, 310, Taiwan

[c]Division of Gyn-Oncology, Department of Obs/Gyn, Chang Gung Memorial Hospital (CGMH), Tao-Yuan 33302, Taiwan

[d]Material and Chemical Research Laboratories, Industrial Technology Research Institute, Hsinchu, 310, Taiwan

[e]Department of Biomedical Sciences, School of Medicine, Chang Gung University (CGU), Tao-Yuan 33302, Taiwan

[f]Department of Physics, Indian Institute of Technology, Kharagpur, 721302, India

[g]S. N. Bose National Centre for Basic Sciences, J D Block, Sector III, Salt Lake City, Kolkata, 106, India

*Corresponding author: E-mail address: sidhu@mail.cgu.edu.tw Tel: +886-3-2118800 ext. 5785

Switching stability by measuring consecutive I-V curves:

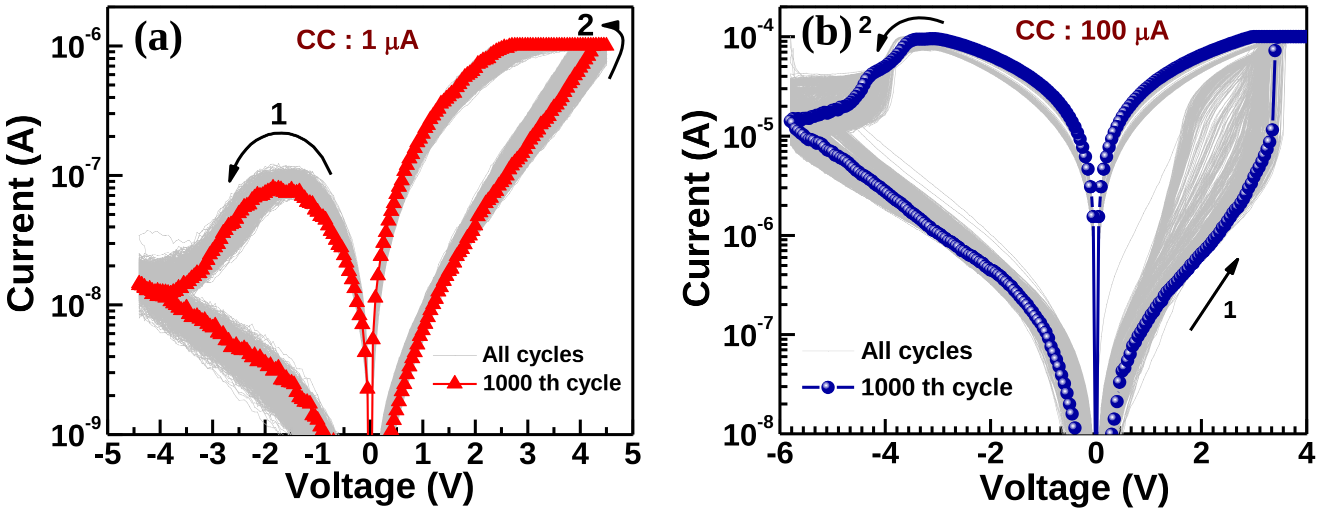

Fig. S1: The W/GeO_x/W RRAM device executes formation-free consecutive I-V switching characteristics of 1000 cycles under current compliances of (a) 1 μA and (b) 100 μA.A small device size of 200 × 200 nm^2 is measured. After 1000 dc cycles, a large acceptable memory window is obtained and this shows excellent switching stability.

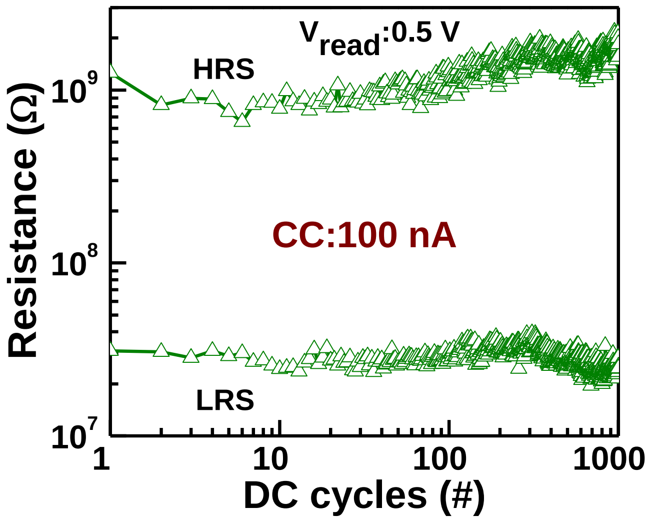

Fig. S2: Resistance vs. dc cycles has been plotted for consecutive 1000 cycles to establish the stability of switching cycles of the W/GeO_x/W RRAM devices. It is worthy to be noted that the device can execute acceptable memory window of approximately 15 with ease at such small CC of 100 nA.

pH sensitivity and drift characteristics:

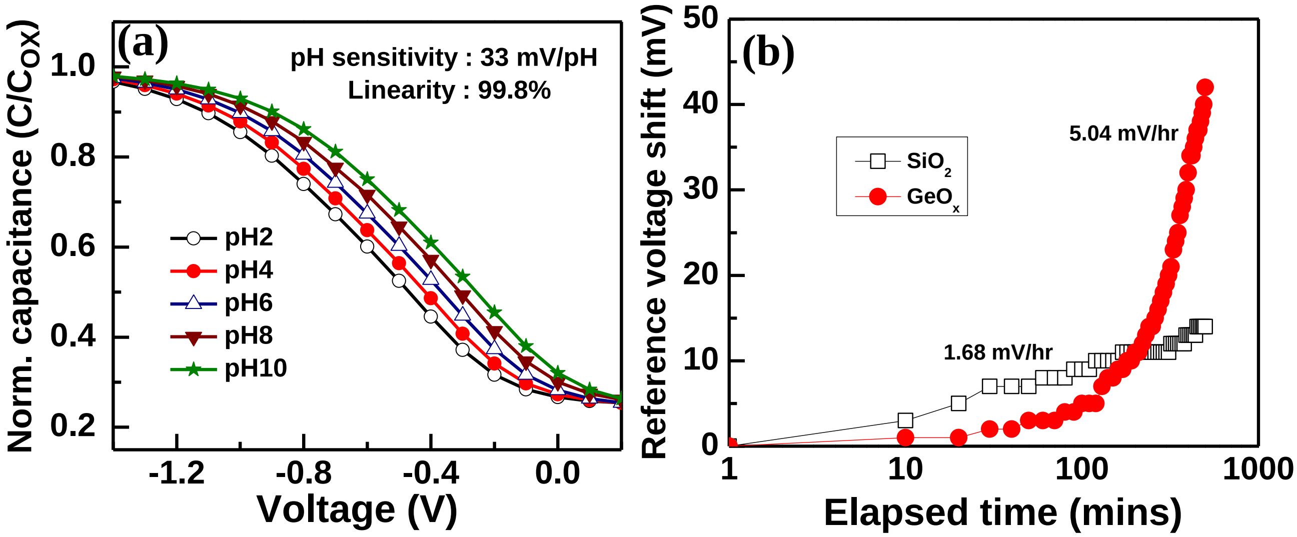

Fig. S3: (a) C-V curves with different pH values from 2 to 10. The GeO_x membrane shows pH sensitivity of 33 mV/pH. A good linearity of 99.8% is obtained. (b) The drift characteristics are shown for both pure SiO_2 and GeO_x membranes. The GeO_x membrane shows slightly higher drift rate than the pure SiO_2 membrane (5.04 mV/hr vs. 1.68 mV/hr) owing to defective GeO_x film. Therefore, a voltage shift of <5 mV is considered for sensing or the reference voltage shift of < 5 mV is noise level.
